# Temporal and clinical heterogeneity of double M-protein multiple myeloma: diagnostic and treatment-emergent phenotypes

**DOI:** 10.3389/fonc.2026.1910010

**Published:** 2026-09-18

**Authors:** Dangui Chen, Fusheng Yao

**Affiliations:** Department of Hematology, Anqing Municipal Hospital, Anqing Medical Center of Anhui Medical University, Anqing, China

**Keywords:** biclonal gammopathy, clonal evolution, double M-protein, immunofixation electrophoresis, minimal residual disease, multiple myeloma, oligoclonal immune reconstitution

## Abstract

**Background:**

Double M-protein multiple myeloma (DMP-MM) is an uncommon phenotype characterized by more than one detectable monoclonal component on serum protein electrophoresis and/or immunofixation electrophoresis. Its clinical significance is uncertain, particularly when a secondary component appears after therapy.

**Methods:**

We retrospectively studied 21 patients with DMP-MM treated at Anqing Municipal Hospital. Patients were classified as diagnostic DMP-MM when double M-protein was present at diagnosis (n=10) or treatment-emergent DMP-MM when a secondary component appeared after therapy (n=11). M-protein patterns, treatment response, progression-free survival (PFS), overall survival (OS), and exploratory Cox models were analyzed from double M-protein recognition. In treatment-emergent cases, pre-treatment M-protein and bone-marrow flow-cytometric minimal residual disease (MRD; sensitivity 10^-5) at secondary-component detection were also evaluated. All analyses were exploratory.

**Results:**

Median age was 66 years (range, 51-84). At initial diagnosis, M-protein was comparable between diagnostic and later treatment-emergent DMP-MM (34.58 vs 24.67 g/L; P = 0.705). In treatment-emergent cases, M-protein decreased from 24.67 to 5.34 g/L at secondary-component recognition (paired P = 0.002), indicating a treatment- and timing-related effect. The newly appearing component had a median concentration of 1.41 g/L (range, 0.39-23.18). PFS events occurred in 8/10 diagnostic and 5/11 treatment-emergent cases; deaths occurred in 6/10 and 4/11, respectively. Two-year PFS was 40.0% (95% CI, 12.3%-67.0%) versus 62.3% (95% CI, 27.7%-84.0%; log-rank P = 0.297), and 2-year OS was 48.0% (95% CI, 16.1%-74.5%) versus 72.7% (95% CI, 37.1%-90.3%; log-rank P = 0.403). Cox estimates were imprecise for PFS (HR 0.557, 95% CI 0.181-1.712) and OS (HR 0.586, 95% CI 0.165-2.084).

**Conclusions:**

Diagnostic and treatment-emergent DMP-MM represent different temporal clinical contexts rather than exchangeable biological groups. Lower M-protein burden at treatment-emergent recognition largely reflects prior therapy and measurement timing. Diagnostic DMP-MM showed a numerically less favorable post-recognition survival trajectory, whereas treatment-emergent secondary M-protein did not show an adverse prognostic signal. Component kinetics, MRD context, and treatment exposure should guide interpretation.

## 1 Introduction

Multiple myeloma (MM) is a clonal plasma-cell malignancy in which secreted monoclonal immunoglobulin, or M-protein, remains central to diagnosis, response assessment, and relapse monitoring. Current diagnostic and response frameworks integrate bone marrow plasma-cell assessment, serum and urine monoclonal protein studies, free light-chain (FLC) testing, imaging, and myeloma-defining events (1, 2). While the traditional clinical model conceptualizes MM as a disease characterized by a single dominant plasma-cell clone producing one monoclonal component, real-world electrophoretic and immunofixation data occasionally reveal two discrete monoclonal bands. This phenomenon is variably referred to as biclonal gammopathy, double monoclonal gammopathy, or double M-protein multiple myeloma (DMP-MM).

DMP-MM presents both diagnostic and biological challenges. The appearance of two bands on serum protein electrophoresis or immunofixation may indicate true biclonal plasma-cell proliferation, two clonally related immunoglobulin variants, class-switch-related immunoglobulin plasticity, a monoclonal immunoglobulin with atypical migration, oligoclonal immune reconstitution following therapy, or analytical interference. Prior studies of biclonal gammopathies suggest that the clinical course may not necessarily be more aggressive than that of monoclonal gammopathy, while molecular interrogation has shown that some double monoclonal gammopathies are clonally related rather than truly independent (3, 4). In addition, apparent double bands may occur with polymeric IgA or other technical phenomena, emphasizing the need to distinguish true double M-protein from pseudo-biclonal patterns (5–7).

Interpretation has become even more complex in the modern treatment era. Anti-CD38 monoclonal antibodies and other therapeutic antibodies can migrate as IgG-kappa bands on immunofixation and mimic endogenous M-protein, while mass spectrometry-based approaches have emerged as more specific tools for separating drug-derived peaks from disease-associated monoclonal proteins (8–11). Consequently, the clinical significance of a second M-protein cannot be deduced solely from the presence of multiple electrophoretic bands. The timing of the appearance of the second component may be particularly significant; a second component present at diagnosis may reflect a different biological state compared to a second component that emerges following effective treatment.

In this study, we analyzed a single-center cohort of 21 patients with DMP-MM and classified them according to the timing of double M-protein recognition. We hypothesized that this timing captures clinically meaningful temporal heterogeneity. We compared initial-diagnosis and recognition-time M-protein burden, immunoglobulin patterns, treatment response, PFS, OS, contemporaneous MRD in treatment-emergent cases, and exploratory Cox models. Our aim was to describe post-recognition clinical trajectories and propose a practical source-trajectory-risk framework, rather than to infer that the two temporal groups are intrinsically biologically equivalent or exchangeable.

## 2 Methods

### 2.1 Study design and setting

This retrospective single-center study was conducted at the Department of Hematology, Anqing Municipal Hospital. Consecutive patients diagnosed with double M-protein multiple myeloma between October 2021 and December 2024 were reviewed. The study was approved by the Ethics Committee of Anqing Municipal Hospital (approval No (2026–110).) and was conducted in accordance with the principles of the Declaration of Helsinki.

### 2.2 Patient selection

Eligible patients were aged 18 years or older and had a confirmed diagnosis of MM with two discrete monoclonal components detected by serum protein electrophoresis and/or immunofixation electrophoresis in the context of bone marrow examination and standard clinical evaluation. Components were categorized as intact immunoglobulin or free-light-chain-only components; a free-light-chain-only secondary event was retained as a distinct operational category and was not assumed to represent an independent second plasma-cell clone. Patients with plasma-cell disorders not requiring anti-myeloma treatment, active non-myeloma malignancies, rheumatic diseases, hepatitis, or suspected daratumumab-related false-positive IgG-kappa bands were excluded. Patients with IgG-kappa myeloma receiving daratumumab were specifically excluded if therapeutic antibody interference could not be reliably ruled out.

### 2.3 Definition of DMP-MM phenotypes

Diagnostic DMP-MM was defined as two monoclonal components detected at initial MM diagnosis before anti-myeloma treatment. Treatment-emergent DMP-MM was defined as the appearance of a secondary monoclonal component during follow-up after treatment initiation. For treatment-emergent cases, the pre-treatment original M-protein, residual original component at secondary-component recognition, newly appearing component, time to emergence, transient persistence, and MRD status were recorded. At first detection of the secondary component, bone marrow was reassessed and MRD was evaluated by flow cytometry with a sensitivity of 10^-5.

### 2.4 Treatment and response assessment

For response analyses, the time origin was defined as the date of double M-protein recognition rather than the date of initial myeloma diagnosis. Treatment was administered according to contemporary institutional practice and current MM treatment principles. All patients received proteasome inhibitor-based therapy. Treatment response after four cycles was evaluated according to International Myeloma Working Group criteria, including no response (NR), partial response (PR), very good partial response (VGPR), complete response (CR), and stringent complete response (sCR). Overall response rate (ORR) was defined as >=PR, and deep responses were summarized as >=VGPR and >=CR.

### 2.5 Outcomes

For survival analyses, the time origin was defined as the date of double M-protein recognition rather than the date of initial myeloma diagnosis. For diagnostic DMP-MM, this corresponded to the initial diagnosis at which both components were present. For treatment-emergent DMP-MM, this corresponded to first detection of the secondary component after treatment initiation. PFS was calculated from this landmark to disease progression, death, or last follow-up, and OS from this landmark to death or last follow-up. This recognition-based landmark removes the pre-recognition immortal-time interval from the treatment-emergent survival clock; however, it does not make the two groups exchangeable because treatment-emergent patients must survive and remain under observation long enough for a secondary component to be detected. Accordingly, survival comparisons were interpreted as descriptive post-recognition trajectories rather than causal prognostic contrasts.

### 2.6 Statistical analysis

Continuous variables were summarized as medians and ranges. Unpaired M-protein comparisons used two-sided exact Mann-Whitney U tests, and the within-patient comparison of M-protein at initial diagnosis versus secondary-component recognition in treatment-emergent cases used a two-sided exact Wilcoxon signed-rank test. Categorical variables were summarized as counts and percentages and compared using Fisher’s exact test or exact tests for multi-category distributions because of small cell counts. Survival was estimated using the Kaplan-Meier method with 95% confidence intervals and compared using log-rank tests. Cox proportional hazards models were exploratory because of the small sample size and limited number of events. All inferential analyses were considered exploratory and hypothesis-generating; P values were two-sided and no adjustment for multiple comparisons was applied.

## 3 Results

### 3.1 Patient characteristics and baseline disease burden

A total of 21 patients with DMP-MM were included, comprising 10 diagnostic DMP-MM and 11 treatment-emergent DMP-MM cases (**Figure 1**). The median age of the overall cohort was 66 years (range, 51-84), and 8 patients (38.1%) were female. Baseline age and sex distribution were comparable between groups. Most patients presented with advanced disease by Durie-Salmon stage (stage III, 17/21), ISS stage III (15/21), and R-ISS stage III (10/21). High-risk FISH abnormalities were present in 12 patients, and all patients received proteasome inhibitor-based treatment. At the double M-protein recognition time point, total measurable M-protein was higher in diagnostic than treatment-emergent DMP-MM (34.58 vs 5.34 g/L; exact P = 0.005; **Table 1**). However, a like-for-like comparison at initial MM diagnosis showed no significant difference between diagnostic DMP-MM and patients who later developed treatment-emergent DMP-MM (34.58 vs 24.67 g/L; exact P = 0.705; **Figure 2A**). Within the treatment-emergent group, total measurable M-protein decreased from 24.67 g/L at initial diagnosis to 5.34 g/L at first secondary-component recognition (paired exact P = 0.002; **Figure 2B**), demonstrating the influence of prior treatment and measurement timing on the recognition-time comparison.

**Figure 1:**
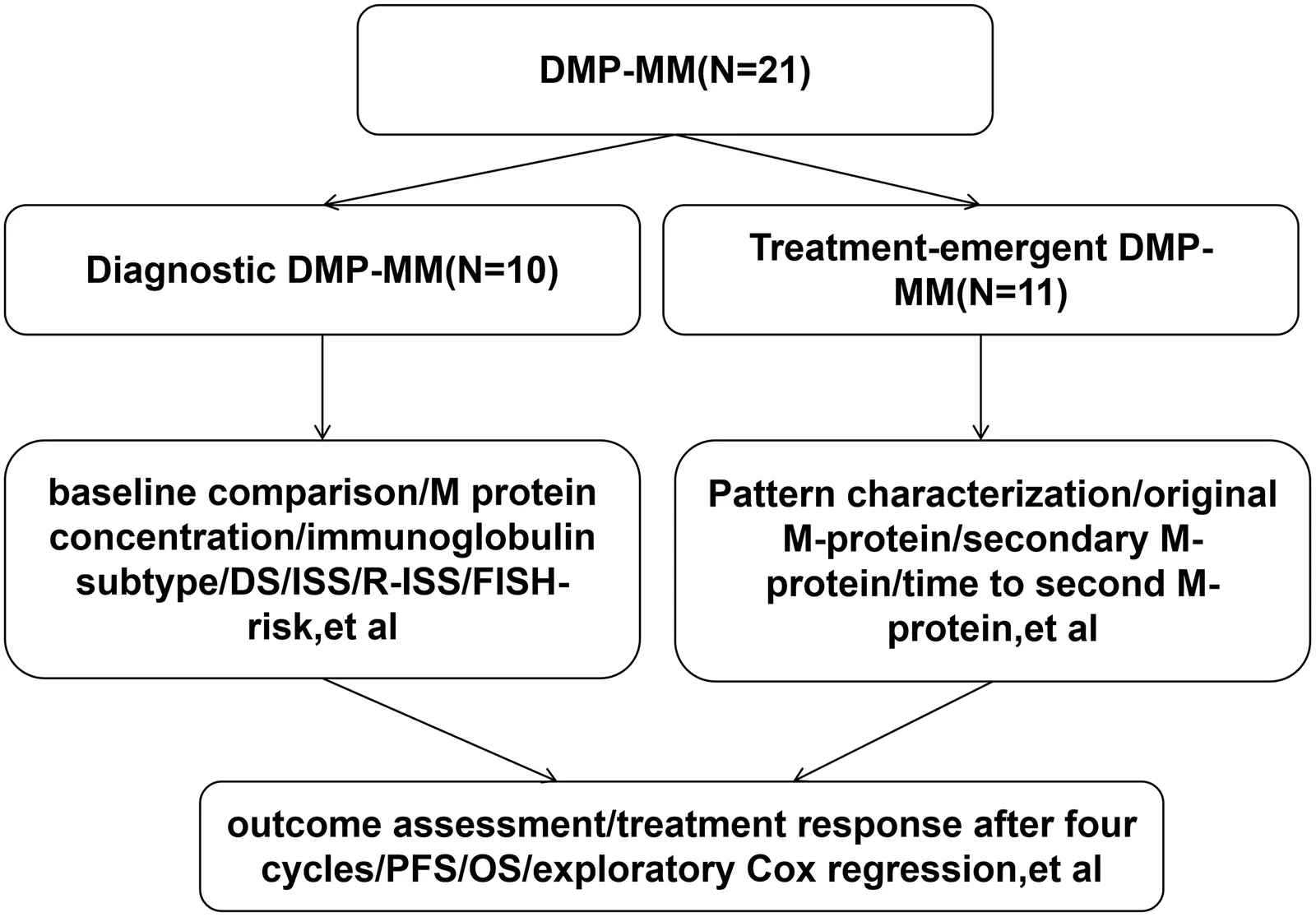
Cohort classification by timing of double M-protein recognition. The 21 included patients were classified as diagnostic DMP-MM (n=10), with two monoclonal components present at initial MM diagnosis before therapy, or treatment-emergent DMP-MM (n=11), with a secondary monoclonal component first detected after treatment initiation. Outcomes were assessed from the double M-protein recognition landmark.

**Table 1:** Baseline characteristics and M-protein burden.

Parameter	Overall	Diagnostic DMP-MM	Treatment-emergent DMP-MM	P value
Female,n	8/21	3	5	0.659
Male,n	13/21	7	6	0.659
Age (year)	66 (51-84)	65 (51-84)	66 (55-78)	0.805
M-protein at DMP recognition (g/L)	9.67 (0.82-89.98)	34.58 (7.16-89.98)	5.34 (0.82-66.88)	0.005
M-protein at initial MM diagnosis (g/L)	24.74 (7.16-89.98)	34.58 (7.16-89.98)	24.67 (8.91-70.98)	0.705
Dominant M-protein				0.705
IgA	6/21	2	4	
IgG	13/21	7	6	
IgM	2/21	1	1	
Secondary component class at DMP recognition				0.198
Intact immunoglobulin	10/21	3	7	
Free light chain only	11/21	7	4	
DS stage I-II	4/21	3	1	0.311
DS stage III	17/21	7	10	
ISS stage I-II	6/21	3	3	1.000
ISS stage III	15/21	7	8	
R-ISS stage I-II	11/21	5	6	1.000
R-ISS stage III	10/21	5	5	
High-risk FISH	12/21	4	8	0.198
Complex chromosome	5/21	4	1	0.149
Auto-SCT	2/21	0	2	0.476
PI-based-Treatment	21/21	10	11	1.000

Data are median (range) or n. For diagnostic DMP-MM, DMP recognition coincided with initial MM diagnosis. For treatment-emergent DMP-MM, initial-diagnosis M-protein was measured before anti-myeloma therapy, whereas recognition-time total measurable M-protein was the sum of quantified monoclonal components at first secondary-component detection. Secondary component class refers to the non-dominant component in diagnostic DMP-MM and the newly appearing component in treatment-emergent DMP-MM. High-risk FISH was defined as t(4;14), t(14;16), t(14;20), gain 1q, or del(17p). DMP-MM, double M-protein multiple myeloma; DS, Durie-Salmon; ISS, International Staging System; R-ISS, Revised ISS; FISH, fluorescence in situ hybridization; Auto-SCT, autologous stem-cell .

**Figure 2:**
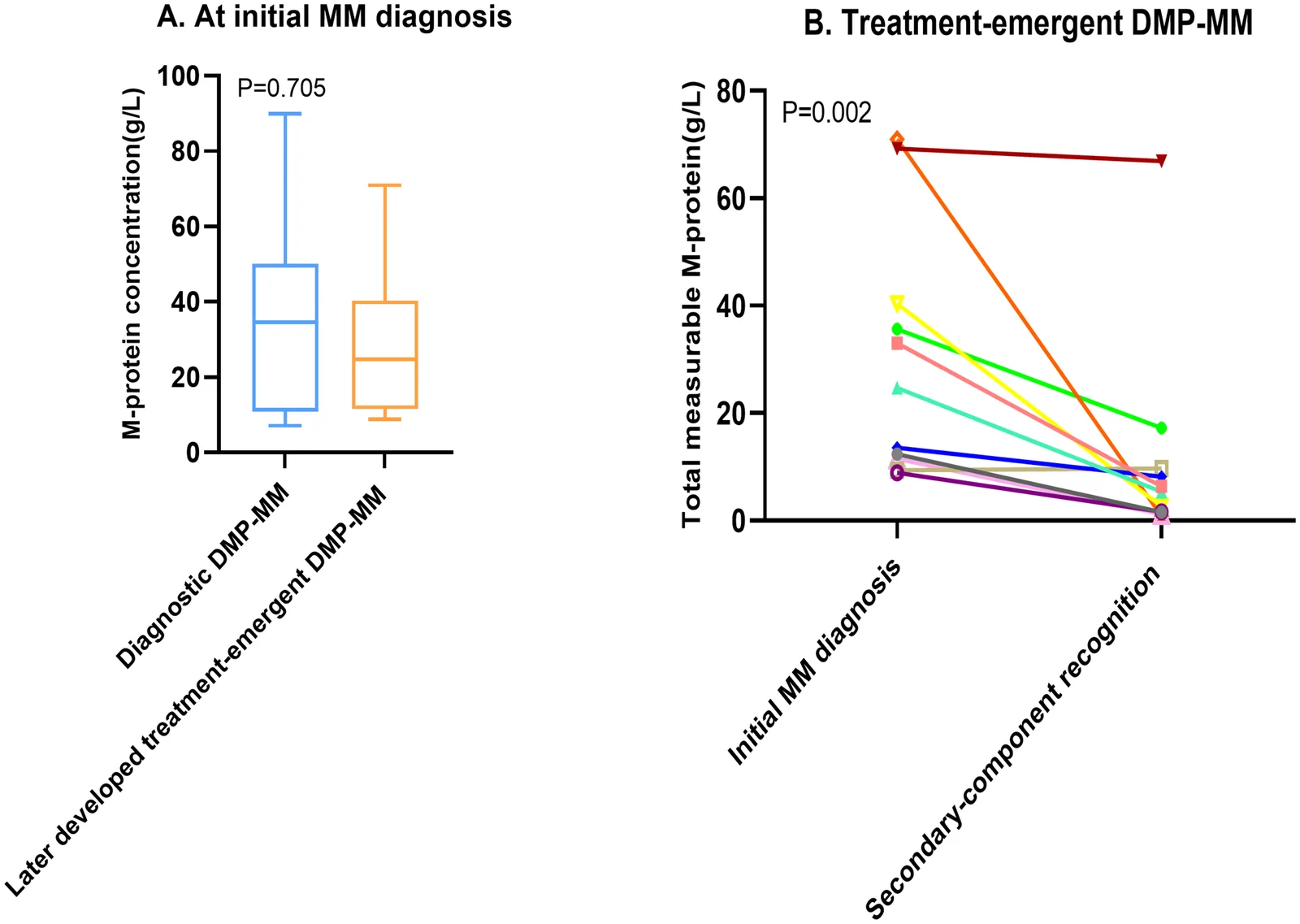
M-protein burden according to measurement timing. **(A)** Total measurable M-protein at initial MM diagnosis was comparable between diagnostic DMP-MM and patients who later developed treatment-emergent DMP-MM (exact P = 0.705). **(B)** In treatment-emergent DMP-MM, total measurable M-protein decreased from initial diagnosis to first recognition of the secondary component (paired exact P = 0.002). Points represent individual patients.

### 3.2 Immunoglobulin patterns and timing of treatment-emergent double M-protein

The first M-protein most frequently involved IgG (13/21), followed by IgA (6/21) and IgM (2/21). Secondary components were heterogeneous and included both intact immunoglobulins and free-light-chain-only components. Among the 11 treatment-emergent cases, the newly appearing component emerged at a median time of approximately 38 months (range, 11-67), and 10/11 secondary components were transiently detected. The newly appearing component had a median concentration of 1.41 g/L (range, 0.39-23.18), while the residual original component had a median concentration of 2.35 g/L (range, 0.33-43.70) at secondary-component recognition. Bone-marrow MRD at this time point was positive in 8/11 and negative in 3/11 patients. In an exploratory analysis, the newly appearing component was numerically higher in MRD-positive than MRD-negative cases (median, 2.23 vs 0.64 g/L; exact permutation P = 0.079), whereas residual original M-protein was higher in MRD-positive cases (5.01 vs 0.51 g/L; exact P = 0.012). These exploratory data suggest that contemporaneous MRD positivity was more strongly related to residual original M-protein burden than to the newly appearing component itself; they do not establish the clonal origin of the secondary component. Patterns included a free-light-chain-only component, heavy-chain switching with the same light chain, light-chain switching with the same heavy chain, and simultaneous heavy/light-chain change (**Table 2**).

**Table 2:** Patterns of treatment-emergent double M-protein.

Patient	Original M-protein	Newly appearing component	Pattern category	Time to second M-protein (months)	Transient	MRD status
1	IgG-kappa	Free kappa	Free light-chain component	32	Yes	Positive
2	IgA-kappa	IgG-kappa	Heavy-chain switch / same light chain	11	Yes	Positive
3	IgA-lambda	Free lambda	Free light-chain component	54	Yes	Positive
4	IgA-lambda	IgG-lambda	Heavy-chain switch / same light chain	42	NO	Positive
5	IgA-kappa	IgG-kappa	Heavy-chain switch / same light chain	13	Yes	Positive
6	IgA-lambda	Free lambda	Free light-chain component	20	Yes	Negativity
7	IgG-lambda	IgG-kappa	Same heavy chain / light-chain switch	38	Yes	Positive
8	IgG-lambda	IgG-kappa	Same heavy chain / light-chain switch	42	Yes	Negativity
9	IgG-kappa	Free kappa	Free light-chain component	67	Yes	Positive
10	IgG-lambda	IgM-kappa	Heavy- and light-chain switch	12	Yes	Negativity
11	IgG-lambda	IgG-kappa	Same heavy chain / light-chain switch	59	Yes	Positive

MRD, minimal residual disease.

### 3.3 Treatment response after four cycles

Treatment response after four cycles was available for all 21 patients. In the diagnostic DMP-MM group, responses included NR in 1 patient (10.0%), PR in 3 (30.0%), VGPR in 4 (40.0%), CR in 1 (10.0%), and sCR in 1 (10.0%). In the treatment-emergent group, responses included NR in 2 patients (18.2%), PR in 1 (9.1%), VGPR in 2 (18.2%), CR in 3 (27.3%), and sCR in 3 (27.3%). The ORR was similar between groups (90.0% vs 81.8%; P = 1.000). Rates of >=VGPR were 60.0% and 72.7%, respectively (P = 0.659), and rates of >=CR were 20.0% and 54.5%, respectively (P = 0.183). Although treatment-emergent DMP-MM showed a numerically higher proportion of deep responses, the overall response distribution did not differ significantly (exact P = 0.502; **Table 3**).

**Table 3:** Treatment response after four cycles according to DMP-MM category.

Variable	Diagnostic DMP-MM (n=10)	Treatment-emergent DMP-MM (n=11)	P value
Overall response distribution			0.411
No response (NR)	1 (10.0%)	2 (18.2%)	
PR	3 (30.0%)	1 (9.1%)	
VGPR	4 (40.0%)	2 (18.2%)	
CR	1 (10.0%)	3 (27.3%)	
sCR	1 (10.0%)	3 (27.3%)	
ORR (>=PR)	9 (90.0%)	9 (81.8%)	1.000
>=VGPR	6 (60.0%)	8 (72.7%)	0.659
>=CR	2 (20.0%)	6 (54.5%)	0.183

The overall response distribution was evaluated using an exact test because of small cell counts; chi-square sensitivity analysis gave P=0.411. ORR, overall response rate; PR, partial response; VGPR, very good partial response; CR, complete response; sCR, stringent complete response.

### 3.4 Survival outcomes and exploratory Cox analysis

Survival analysis was performed from the time of double M-protein recognition. PFS events occurred in 8/10 diagnostic and 5/11 treatment-emergent patients. Median PFS was 24 and 30 months, respectively. Two-year PFS was 40.0% (95% CI, 12.3%-67.0%) versus 62.3% (95% CI, 27.7%-84.0%), with no statistically significant difference (log-rank P = 0.297). Deaths occurred in 6/10 diagnostic and 4/11 treatment-emergent patients. Two-year OS was 48.0% (95% CI, 16.1%-74.5%) versus 72.7% (95% CI, 37.1%-90.3%), again without a statistically significant difference (log-rank P = 0.403; **Figure 3**). Exploratory Cox models yielded imprecise estimates for treatment-emergent versus diagnostic DMP-MM for PFS (HR 0.557, 95% CI 0.181-1.712; P = 0.307) and OS (HR 0.586, 95% CI 0.165-2.084; P = 0.409). Response depth was not entered as a formal Cox covariate because CR/sCR patients had no observed PFS or OS events, producing complete separation and unstable estimates (**Table 4**).

**Figure 3:**
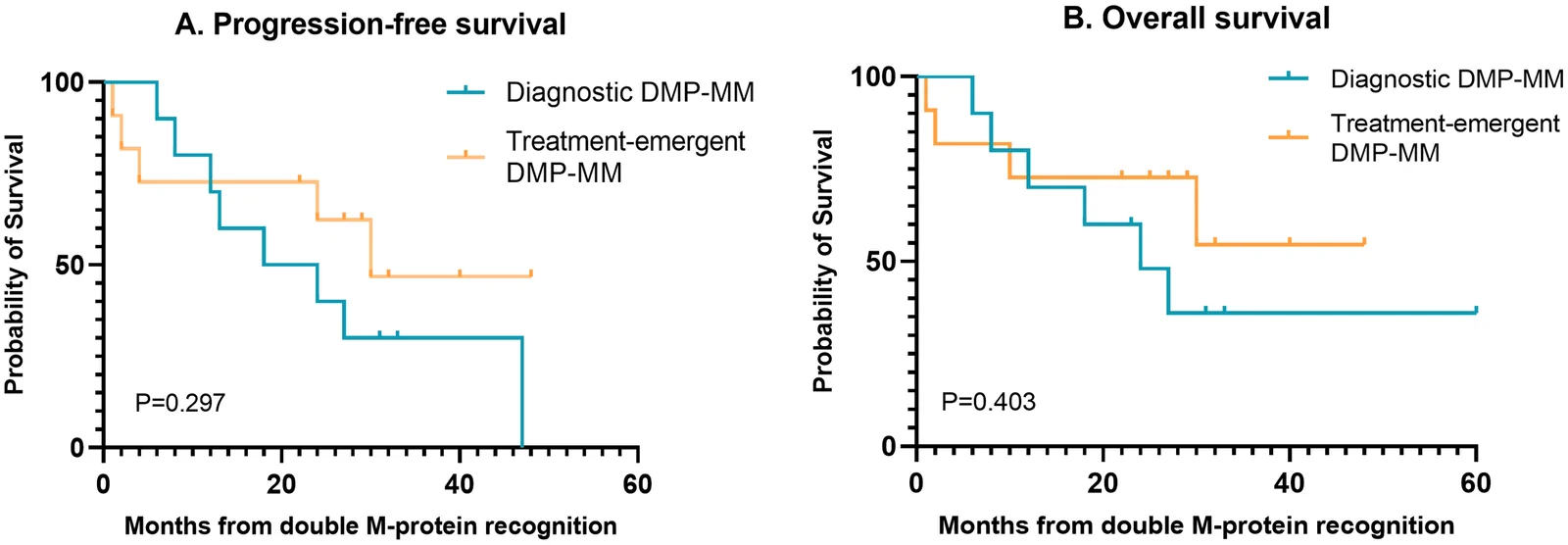
Kaplan-Meier analysis from the time of double M-protein recognition. **(A)** Progression-free survival; **(B)** overall survival. Number-at-risk tables and censoring marks are shown. For diagnostic DMP-MM, the time origin was the date of initial diagnosis at which both components were present. For treatment-emergent DMP-MM, the time origin was the date when the secondary component was first detected after treatment initiation. The curves describe post-recognition trajectories and should not be interpreted as a causal comparison of exchangeable groups.

**Table 4:** Cox proportional hazards analysis for PFS and OS.

Outcome	Covariate	Comparison	HR	95% CI	P value
PFS	Group	Treatment-emergent vs Diagnostic	0.557	0.181-1.712	0.307
OS	Group	Treatment-emergent vs Diagnostic	0.586	0.165-2.084	0.409
PFS	Response depth	CR/sCR vs <CR	NE	NE	NE
OS	Response depth	CR/sCR vs <CR	NE	NE	NE

Cox models used Breslow handling for ties and were exploratory/hypothesis-generating because of the small number of events. The HR for group compares treatment-emergent DMP-MM with diagnostic DMP-MM. Response depth was not entered as a formal Cox covariate because CR/sCR patients had no PFS or OS events, producing complete separation and unstable estimates. HR, hazard ratio; NE, not estimable.

### 3.5 Representative immunofixation finding

A representative immunofixation electrophoresis pattern demonstrated double M-protein bands with IgG-lambda and IgA-kappa components (**Figure 4**). This illustrates the diagnostic basis for DMP-MM and emphasizes the importance of immunofixation in defining heavy-chain and light-chain pairing.

**Figure 4:**
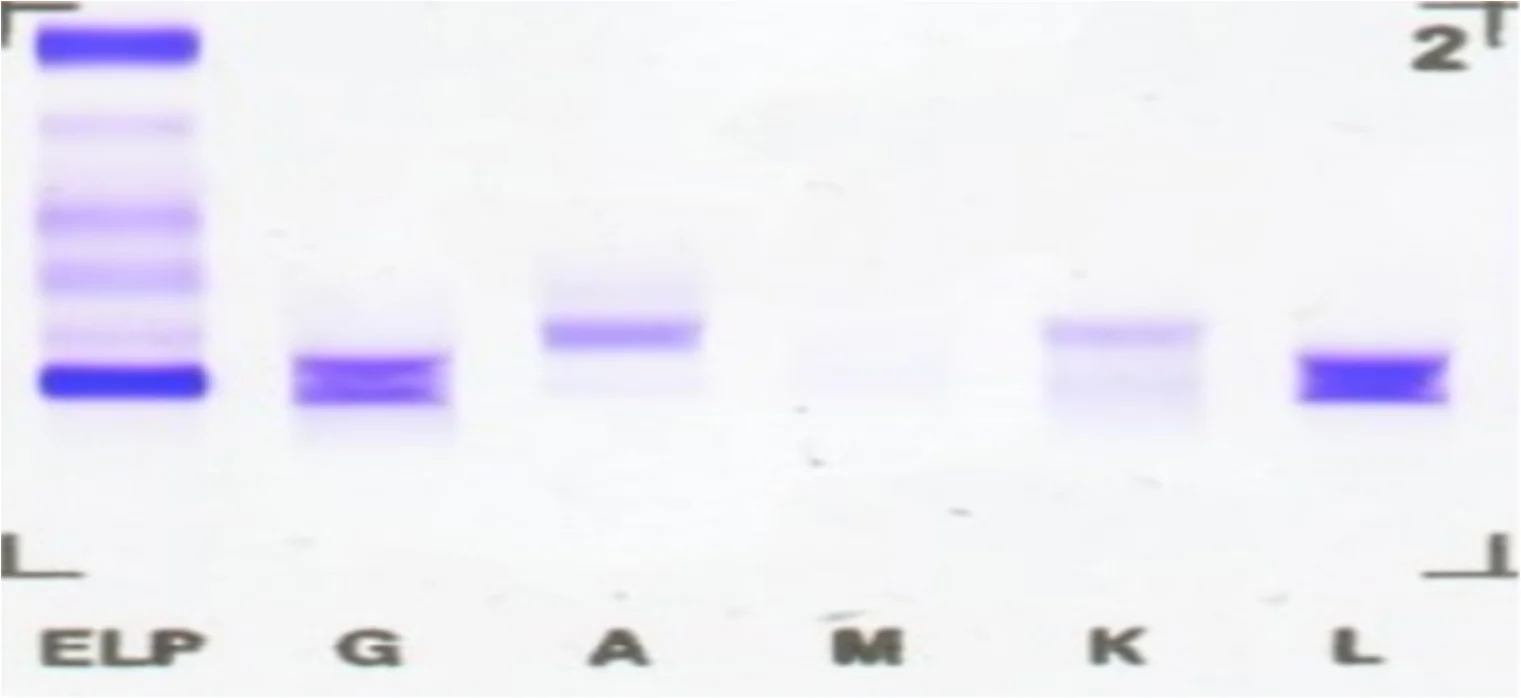
Representative immunofixation electrophoresis showing double M-protein bands with IgG-λ and IgA-κ components.

## 4 Discussion

In this single-center retrospective study of 21 patients, we used the timing of double M-protein recognition to describe diagnostic and treatment-emergent DMP-MM as two temporal clinical phenotypes. The revised analyses emphasize that these groups arise in different treatment contexts and are not exchangeable. At initial MM diagnosis, M-protein burden was comparable between groups, whereas the treatment-emergent group had substantially lower M-protein by the time the secondary component was recognized after therapy. Post-recognition PFS and OS were numerically less favorable in diagnostic DMP-MM, but confidence intervals were wide and between-group differences were not statistically significant. These findings therefore describe heterogeneous clinical trajectories rather than proving distinct intrinsic biological states.

The recognition-time M-protein difference requires careful interpretation. Diagnostic patients were measured at untreated presentation, whereas treatment-emergent patients were measured only after prior anti-myeloma therapy. Consistent with this, total measurable M-protein at initial MM diagnosis did not differ significantly between groups, while treatment-emergent patients showed a marked within-patient decline by secondary-component recognition. Thus, the lower recognition-time M-protein in treatment-emergent DMP-MM is largely a treatment- and timing-related observation and should not be used as evidence of an intrinsically lower-secretory or less aggressive biological phenotype. M-protein remains a useful marker of measurable secretion, but it is not necessarily equivalent to tumor mass or biological aggressiveness; imaging-biomarker data likewise suggest that M-protein and FLC levels may incompletely capture tumor volume in some MM contexts (12).

A clinically important observation is that a treatment-emergent secondary component did not show an adverse post-recognition prognostic signal in this cohort. Such a component appeared 11–67 months after treatment initiation and was transient in 10/11 cases. In addition, contemporaneous marrow MRD provided important context: MRD-positive cases tended to have higher newly appearing component levels and had higher residual original M-protein levels than MRD-negative cases. These findings support a contextual interpretation that includes oligoclonal immune reconstitution, persistence or expansion of a previously minor clone, immunoglobulin plasticity, light-chain escape, and true clonal progression as alternative explanations. Because clonality was not established molecularly, neither low-level MRD-negative bands nor higher-level MRD-positive bands can be assigned a specific mechanism from these data alone.

The distinction between true biclonality and electrophoretic multiplicity is central to interpretation. Molecular studies have shown that double monoclonal gammopathies may be clonally related, with different heavy-chain isotypes sharing immunoglobulin gene rearrangements (4). Conversely, apparent double bands may result from polymeric immunoglobulin behavior or technical factors, particularly in IgA-associated gammopathies. Free-light-chain-only secondary events also warrant separate consideration because they may reflect light-chain escape or other post-treatment phenomena rather than a new intact-immunoglobulin clone. These considerations support use of the descriptive term double M-protein MM rather than biclonal MM unless clonal independence is demonstrated by molecular or cellular methods.

The modern treatment era adds another layer of complexity. Therapeutic monoclonal antibodies can mimic endogenous M-protein on electrophoretic assays, particularly anti-CD38 antibodies such as daratumumab. This issue has led to the development of Hydrashift-based assays and mass spectrometry approaches to distinguish drug-related bands from disease-associated M-protein (8, 9). Although our cohort excluded suspected daratumumab-related IgG-kappa interference, this consideration is essential for future DMP-MM studies. In patients receiving antibody-based therapies, a newly appearing M-protein band should not be classified as treatment-emergent DMP-MM without careful assessment of drug migration and baseline immunoglobulin identity.

The survival analyses are hypothesis-generating. Although PFS and OS were numerically longer after recognition in the treatment-emergent group, the groups are intrinsically non-exchangeable: treatment-emergent patients must survive and remain under treatment or observation long enough for a secondary component to be detected. The recognition landmark removes the pre-recognition immortal-time interval from the survival clock but does not eliminate this selection. Accordingly, the numerical survival difference should not be interpreted as evidence that treatment-emergent DMP-MM is intrinsically benign or confers a survival advantage. Prior work on biclonal gammopathies has likewise suggested that outcomes may be comparable with monoclonal counterparts and that a new monoclonal protein after therapy need not carry adverse implications (3). In a separate retrospective MM cohort, Zhang et al. reported that ANA and particularly SSA autoantibody positivity were associated with shorter PFS (13). Although autoantibodies are biologically distinct from secondary monoclonal components, this study further illustrates that abnormal antibody-related signals in MM can have context-dependent prognostic associations and should not be treated as interchangeable phenomena.

The survival analyses are hypothesis-generating. Although PFS and OS were numerically longer after recognition in the treatment-emergent group, the groups are intrinsically non-exchangeable: treatment-emergent patients must survive and remain under treatment or observation long enough for a secondary component to be detected. The recognition landmark removes the pre-recognition immortal-time interval from the survival clock but does not eliminate this selection. Accordingly, the numerical survival difference should not be interpreted as evidence that treatment-emergent DMP-MM is intrinsically benign or confers a survival advantage. Prior work on biclonal gammopathies has likewise suggested that outcomes may be comparable with monoclonal counterparts and that a new monoclonal protein after therapy need not carry adverse implications (3). In a separate retrospective MM cohort, Zhang et al. reported that ANA and particularly SSA autoantibody positivity were associated with shorter PFS (13). Although autoantibodies are biologically distinct from secondary monoclonal components, this study further illustrates that abnormal antibody-related signals in MM can have context-dependent prognostic associations and should not be treated as interchangeable phenomena.

This study has several limitations. The sample size was small, reflecting the rarity of DMP-MM, and the retrospective design limits causal inference and statistical precision. Molecular clonality assessment, immunoglobulin gene sequencing, and mass spectrometry-based clonotyping were not available; therefore, the biological source of a secondary component could not be established directly. Bone-marrow MRD was assessed at first secondary-component detection in treatment-emergent cases, but serial MRD assessment was not uniform at all other time points. All patients received proteasome inhibitor-based therapy, limiting evaluation of regimen-specific effects. Most importantly, the diagnostic and treatment-emergent groups were not exchangeable. Using double M-protein recognition as the survival landmark removes the pre-recognition immortal-time interval but cannot remove the selection inherent in surviving and remaining under observation until a secondary component is detected. All inferential findings should therefore be regarded as exploratory and require validation in larger prospective cohorts.

Future studies should prospectively enroll DMP-MM patients and classify cases by timing, paired heavy-chain/light-chain identity, treatment exposure, molecular clonality, and serial M-protein kinetics. Serum mass spectrometry and immunoglobulin gene sequencing could provide a direct method to determine whether two M-proteins are clonally related, independent, drug-derived, or oligoclonal. Integrating these approaches with marrow MRD and immune-repertoire profiling may clarify whether treatment-emergent DMP-MM reflects humoral immune recovery, subclonal evolution, or both.

## 5 Conclusion

Using double M-protein recognition as the survival landmark defines prognosis from the clinically relevant moment at which DMP-MM is recognized and removes the pre-recognition immortal-time interval from treatment-emergent follow-up; it does not make the two temporal groups exchangeable. At initial MM diagnosis, M-protein burden was comparable between groups, while the lower burden at treatment-emergent recognition largely reflected prior therapy and measurement timing. From the recognition landmark, diagnostic DMP-MM showed a numerically less favorable PFS/OS trajectory, whereas treatment-emergent secondary M-protein did not show an adverse prognostic signal; however, these small-cohort differences were not statistically significant and should not be interpreted causally. The presence of double M-protein alone should therefore not be regarded as an indicator of aggressive disease. Timing of emergence, component-specific kinetics, contemporaneous marrow MRD, treatment exposure, and source attribution should guide clinical interpretation.

## Data Availability

The original contributions presented in the study are included in the article/supplementary material. Further inquiries can be directed to the corresponding author.
